# Inoperable or incompletely resected craniofacial osteosarcoma treated by particle radiotherapy

**DOI:** 10.3389/fonc.2022.927399

**Published:** 2022-09-23

**Authors:** Katharina Seidensaal, Matthias Dostal, Jakob Liermann, Sebastian Adeberg, Fabian Weykamp, Maximillian P. Schmid, Christian Freudlsperger, Jürgen Hoffmann, Ivar Hompland, Klaus Herfarth, Jürgen Debus, Semi B. Harrabi

**Affiliations:** ^1^ Department of Radiation Oncology, Heidelberg University Hospital, Heidelberg, Germany; ^2^ Heidelberg Institute of Radiation Oncology (HIRO), Heidelberg, Germany; ^3^ National Center for Tumor diseases (NCT), Heidelberg, Germany; ^4^ Heidelberg Ion-Beam Therapy Center (HIT), Department of Radiation Oncology, Heidelberg University, Heidelberg, Germany; ^5^ Clinical Cooperation Unit Radiation Oncology, German Cancer Research Center (DKFZ), Heidelberg, Germany; ^6^ Department of Radiation Oncology, Comprehensive Cancer Center, Medical University of Vienna, Vienna, Austria; ^7^ Department of Oral and Maxillofacial Surgery, Heidelberg University Hospital, Heidelberg, Germany; ^8^ Department of Oncology, Norwegian Radium Hospital, Oslo University Hospital, Oslo, Norway; ^9^ German Cancer Consortium (DKTK), Heidelberg, Germany

**Keywords:** craniofacial osteosarcoma, proton radiotherapy, carbon ion radiotherapy, survival outcome, particle radiotherapy

## Abstract

**Background:**

To report survival of craniofacial osteosarcoma patients treated by particle radiotherapy.

**Methods:**

Between January 2010 and December 2021, 51 patients with primary (*N* = 35) or recurrent (*N* = 16) inoperable or incompletely resected craniofacial osteosarcoma were treated. In most cases, intracranial infiltration (59%) and macroscopic tumor on MRI/CT (75%) were present. Thirteen had a secondary osteosarcoma (25%). Treatment concepts included combined ion beam radiotherapy (CIBRT, *N* = 18), protons only (*N* = 3), carbon ions only (*N* = 12), IMRT with a carbon ion boost (*N* = 5), and carbon ion re-irradiation (*N* = 13). Eighty percent (N = 41) received additionally chemotherapy, most frequently EURAMOS-1 (47%) or EURO-B.O.S.S. (18%).

**Results:**

The median age was 38, and all patients finished treatment predominantly as outpatients (*N* = 44). Information on overall survival was available for N = 49 patients. The median follow-up of the survivors was 55 months. For the whole cohort 1-, 2-, 3-, and 5-year overall survival (OS) was 82.8%, 60.4%, 55.2%, and 51.7%, respectively. Those treated by CIBRT (N = 17) demonstrated a superior OS with 92.9% after 1 and 2 years and 83.6% after 3 and 5 years. The median clinical target volume (CTV) was 192.7 and 95.2 cc for the primary and boost plan, respectively. CIBRT, primary diagnosis, age ≤40a, and no macroscopic residual tumor were associated with improved survival in univariate analysis (*p* = 0.006, *p* = 0.004, *p* = 0.002, *p* = 0.026, respectively), while any foregoing resection compared to biopsy was not identified as a prognostic factor. CIBRT and no macroscopic residual tumor were confirmed as independent predictors of OS on multivariate analysis (HR = 0.107, 95% CI = [0.014, 0.797], *p* = 0.029 and HR = 0.130, 95% CI = [0.023, 0.724], *p* = 0.020, respectively). No acute toxicity > grade III was observed.

**Conclusion:**

CIBRT shows promising results for patients with inoperable or incompletely resected craniofacial osteosarcoma.

## Introduction

Osteosarcoma is the most prevalent malignant bone tumor in children and adolescents, arising mostly at the distal femur and the proximal tibia. It is characterized by an early formation of metastases (1). Craniofacial osteosarcoma (CFOS) represents only 10% of all osteosarcoma and shows significant differences in the clinical course compared to osteosarcoma of the trunk or extremity. The typical age at first diagnosis is in the third and fourth decades compared to the second decade of life in case of extracranial osteosarcoma. Furthermore, there is a lower propensity for distant metastases and the 5-year survival of CFOS is superior compared to extracranial osteosarcoma, but the mortality due to the difficulty of obtaining local control is higher (2).

Craniofacial bone shows different characteristics compared to extracranial bone in regard to turnover, remodeling, and the expression of differentiation markers. Furthermore, a lower activation of the Hedgehog signaling pathway was shown recently for CFOS. To date, the molecular mechanisms behind these clinical differences are not fully understood (3).

Primary surgery is recommended for most cases, and complete resection is pivotal for local control. The jaw is the most common location, but especially in cases with involvement of the skull, orbit, and paranasal sinuses, incomplete resection, recurrence, and progression are frequent. The role of radiotherapy (RT) for inoperable and incompletely resected CFOS is not firmly established, but the ESMO-EUROCAN Guidelines recommend discussing RT in cases of positive resection margins or inoperability (4). The role of multiagent chemotherapy, which is firmly established for other locations and has led to a tremendous improvement of survival (<20% vs. >60%), is under debate, and the results are so far conflicting (5). Nonetheless, the current ESMO-EURACAN Guidelines recommend to handle high-grade CFOS according to the regimens for other locations (4).

We have previously presented the results of the prospective phase I/II OSCAR trial combining proton and carbon ion radiotherapy (CIBRT) for inoperable osteosarcoma implemented into the standard chemotherapy regimens as EURAMOS-1 and COSS96, which were promising especially for the small cohort of CFOS (*N* = 6) (6). Herein, we present a larger cohort of patients with CFOS treated in analogy to the OSCAR trial, an update of the OSCAR trial participants (previously reported until May 2019), and patients treated with other regimens of particle therapy.

## Materials/methods

### Data collection

The study was approved by the Ethics committee of the University of Heidelberg. Data were obtained from retrospective review of medical records. Survival data of German residents were updated by the resident’s registration offices and the German Cancer registry.

### Study cohort

The cohort (*N* = 51) consisted of 24 female and 27 male patients. The most common histology was osteoblastic and chondroblastic osteosarcoma (28% and 26%, respectively). Four patients presented with distant metastasis at the time of radiotherapy.

Radiotherapy was performed for primary disease in 69% and for recurrent disease in 31% of cases. Macroscopic residual tumor was present in 75% of cases. Most tumors involved multiple anatomical regions (**Table 1**) of those 59% with intracranial extension.

**Table 1:** Patient characteristics.

	*N = 51*	Range or %
**Median age at radiotherapy**	38	9-78
**Median age at first diagnosis**	35	9-78
**Median time from ED to RT (months)**	8	1-109
**Gender**		
Female	24	47
Male	27	53
**Histology**		
Osteoblastic	14	28
Chondroblastic	13	26
Osteo- and chondroblastic	3	6
Osteo-, chondro-, and fibroblastic	1	2
Low-grade central	1	2
Parosteal	1	2
Fibroblastic	1	2
Extraosseous	1	2
Missing subtype information	16	31
**Anatomically involved region**		
Maxillary sinus, nasal cavity, orbit	9	
Orbit with intracranial infiltration	6	
Mandible	5	
Petrous bone	4	
Maxillary sinus	4	
Maxillary sinus, orbit	4	
Temporal	2	
Occipital	2	
Parieto-temporal	2	
Frontal	2	
Temporo-occipital	2	
Petrous bone, orbit, sphenoid bone	2	
Maxillary sinus, nasal cavity, pterygopalatine fossa	1	
Sphenoid, ethmoid, paranasal sinuses	1	
Maxillary sinus, mandible, nasal cavity, temporal fossa	1	
Base of the mouth	1	
Maxillary sinus, mandible	1	
Cervical/paravertebral	1	
Sphenoid bone, zygomatic bone	1	
**Intracranial infiltration**		
Yes	30	59
No	21	41
**Secondary Osteosarcoma**		
No	38	75
Yes	13	25
**Previous diagnosis (secondary OS)**		
Retinoblastoma	5	
Ependymoma	1	
Pineoblastoma	1	
Pituitary gland adenoma	1	
Inverted papilloma	1	
Tonsillary carcinoma	1	
Nasopharyngeal carcinoma	1	
Adenoid cystic carcinoma	1	
Pleomorphic sarcoma	1	
**Median time from RT to secondary OS (years)**	18	5-28
**Median total dose of previous RT (Gy)**	50	45-70
**Grading**		
G1	3	6
G2	5	10
G2-3	1	2
G3	17	33
High grade	14	28
Low grade	2	4
Missing	9	17
**Boost plan clinical target volume**		
N	29	
Median, range in ccm	95.2	19.9 - 339.4
**Primary plan clinical target volume**		
N	49	
Median, range in ccm	192.7	8.4 - 1315.3
**Primary/recurrence**		
Primary	35	69
Recurrence	16	31
**Residual tumor**		
Macroscopic (MRI/CT)	38	75
Microscopic	13	25
**Biopsy or surgery before treatment**		
Biopsy	16	31
Any preceding resection	35	69
**Distant metastases at beginning of hadron therapy**		
Yes	4	8
No	47	92
**Treatment concept**		
OSCAR trial	6	12
^1^H: 54 GyRBE in 27 Fx, ^12^C: 18 GyRBE in 6 Fx	6	
OSCAR analog	12	24
^1^H: 54 GyRBE in 27 Fx, ^12^C: 18 GyRBE in 6 Fx	12	
Proton only	3	6
^1^H: 60 GyRBE in 30 Fx	2	
^1^H: 66 GyRBE in 33 Fx	1	
Carbon ion only	12	24
^12^C: 60 GyRBE in 20 Fx	3	
^12^C: 66 GyRBE in 22 Fx	9	
Carbon ion re-irradiation	13	26
^12^C: 63 GyRBE in 21 Fx	1	
^12^C: 60 GyRBE in 20 Fx	6	
^12^C: 54 GyRBE in 18 Fx	1	
^12^C: 51 GyRBE in 17 Fx	2	
^12^C: 18 GyRBE in 6 Fx + 54 Gy IMRT	1	
Carbon ion boost and IMRT	5	10
^12^C: 24 GyRBE in 8 Fx + 54 Gy IMRT	1	
^12^C: 24 GyRBE in 8 Fx + 40 Gy IMRT	1	
^12^C: 24 GyRBE in 8 Fx + 42 Gy IMRT	1	
^12^C: 18 GyRBE in 6 Fx + 54 Gy IMRT	1	
^12^C: 18 GyRBE in 6 Fx + 45 Gy IMRT	1	
**Chemotherapy protocol**		
No chemotherapy	10	20
EURAMOS-1 (thereof before recurrence)	24 4	47
EURO-B.O.S.S. (thereof before recurrence)	9 1	18
ICE	2	4
OS 2006-API/AI	1	2
CWS	2	4
Sarcoma 13 OS 2016	1	2
**Outpatient/inpatient treatment**		
Outpatient	44	86
Hospitalized	7	14

Thirteen patients had a secondary osteosarcoma after previous RT for retinoblastoma (*N* = 5), ependymoma, pineoblastoma, inverted papilloma, tonsil carcinoma, nasopharyngeal carcinoma, adenoid cystic carcinoma, or pleomorphic sarcoma (each N=1). The median time from first RT until the treatment of secondary osteosarcoma was 18 years (5–28 years). The median dose of previous RT was 50 Gy (45–70 Gy).

Due to the rarity of the disease, the cohort consisted of patients from eight different countries. In part, follow-up was performed by the treating oncologist and reported to our center, including the provision of images and reports.

### Treatment regimen and facility

CIBRT consisted of a proton primary plan of 54 Gy relative biological effectiveness (RBE) in 27 fractions and a carbon ion boost plan (BP) of 18 Gy (RBE) in six fractions—33 fractions in total. Six patients were treated within the OSCAR trial, and additional 12 patients were treated according to this regimen. Three patients were treated with protons only with a total dose of 60/66 Gy(RBE) in 2 Gy(RBE) single doses. Carbon ion radiotherapy was performed with 3 Gy(RBE) single doses. The total dose of the carbon ion only subgroup (*N* = 12) was 60 Gy(RBE) in three and 66 Gy(RBE) in nine cases. Carbon ion re-irradiation was performed with a total dose of 51–63 Gy(RBE). Five patients received a carbon ion boost of 18–24 Gy(RBE) combined with an intensity-modulated radiation therapy (IMRT) base plan (40–54 Gy) (**Table 1**). The RBE dose of carbon ions was calculated using the LEM1 model (7).

Treatment was administered at the Heidelberg Ion-Beam Therapy Centre (HIT, N = 49) and at the Marburg Ion-Beam Therapy Centre (MIT, N = 2) in active raster scanning technique (8, 9). During treatment, patients were monitored weekly. Chemotherapy was administered before and/or after radiotherapy in 80% of patients, mostly according to the protocols EURAMOS-1 (47%) or EURO-B.O.S.S (18%) (10). No patient received chemotherapy concomitant with RT. Four patients were treated according to the protocol EURAMOS-1, and one treated according to EURO-B.O.S.S had a recurrence after chemotherapy and before CIBRT (**Table 1**).

### Target volume definition

**Figure 1:**
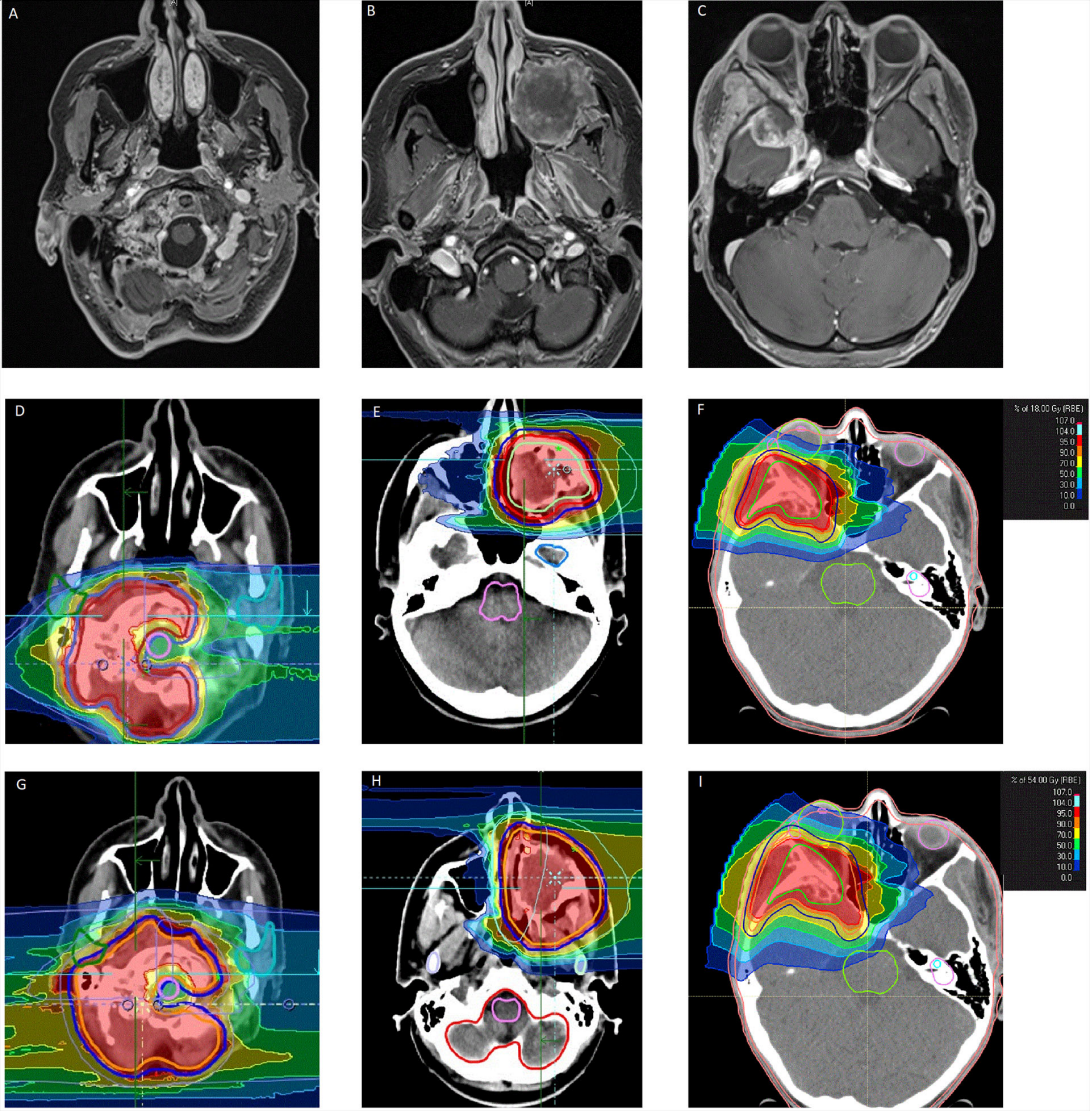
Examples of craniofacial osteosarcoma treated by combined ion-beam radiotherapy. **(A–C)** Representative contrast-enhanced T1 MRI sequences of inoperable craniofacial osteosarcoma. **(D–F)** Target volume delineation and dose distribution of the according carbon ion boost (18 GyRBE in six fractions). **(G–I)** Target volume delineation and dose distribution of the according proton base boost (54 GyRBE in 27 fractions).

CIBRT: The gross tumor volume (GTV) was delineated as all visible macroscopic tumors on the basis of contrast-enhanced MRI and CT at the time of presentation. To obtain the clinical target volume (CTV) for the boost volume, a safety margin of 3 mm (up to 7 mm) was added. In cases without macroscopic residual tumor, the delineation of the boost was guided by the initial tumor extension. The CTV of the primary proton plan comprised the CTV boost and an additional margin of 2 cm added to the GTV. The primary plan CTV included the initial tumor extension after previous subtotal resection (**Figure 1**).

Other regimens: Treatment by carbon ions only was performed without a boost in most cases. A margin of 1–2 cm was added to the GTV. Re-irradiation was performed with smaller margins of 5–10 mm.

The margins were adapted respecting anatomic boundaries or previously uninvolved dislocated organs at risk. The planning target volume (PTV) was acquired by an isotropic 3-mm margin.

### Statistical analysis

Local progression-free survival (PFS) and overall survival (OS) were analyzed by means of a Kaplan–Meier curve with report of 1-,2-, 3-, and 5-year survival rates. Additionally, stratified Kaplan–Meier curves were plotted for subgroup factors and tested by the log-rank test. Multivariate analysis was performed by Cox proportional hazard regression. Hazard ratios (HR) with 95% CI were provided. P-values <0.05 were considered significant.

Analyses were done using SPSS V 27.

## Results

The median age at RT was 38 years (range: 9–78). The median CTV was 192.7 cc (range: 8.4–1315.2cc) for the primary plan and 95.2 cc (range: 19.9–339.4) for the boost plan, respectively (**Table 1**).

**Figure 2:**
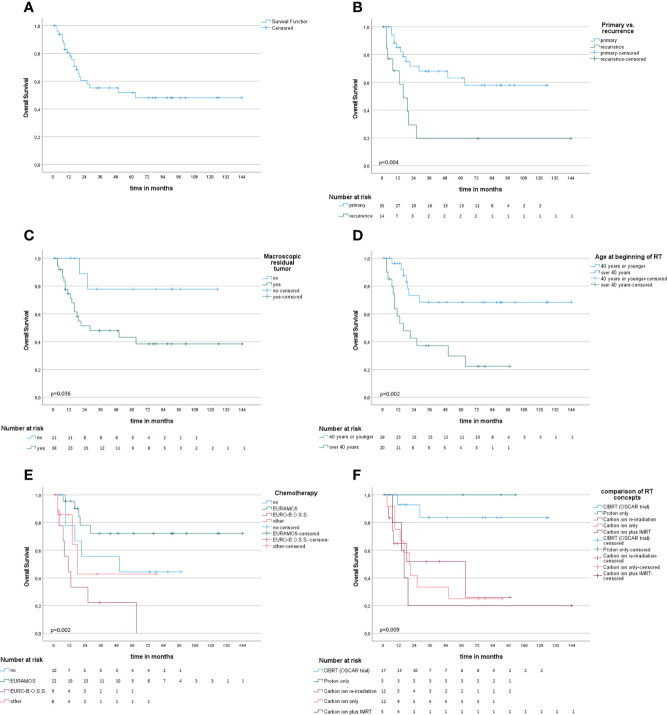
Kaplan–Meier survival analysis **(A)** and univariate subgroup analysis **(B–F)**. **(A)** Overall survival probability. **(B)** Radiotherapy at the time of primary diagnosis compared to recurrent disease is associated with improved survival. **(C, D)** Macroscopic residual tumor on MRI or CT and age above 40 years at beginning of RT are associated with impaired OS. **(E)** Comparison of different chemotherapy concepts. **(F)** Comparison of different concepts of particle radiotherapy.

Survival information was available for 49 patients; 28 patients (57%) were alive at the time of this analysis, and the median follow-up (FU) of the survivors was 55 months. For the whole cohort, OS after 1, 2, 3, and 5 years was 82.8%, 60.4%, 55.2%, and 51.7%, respectively (**Figure 2A**). For those who were treated for primary disease, the OS after 1, 2, 3, and 5 years was 85.2%, 71.6%, 68.1%, and 63.2%, respectively, and significantly better (p = .004) compared to recurrent disease 68.4% and 29.3% after 1 and 2 years (**Figure 2B**). Regarding the different treatment regimens, patients treated with the CIBRT concept of the OSCAR trial (*N* = 17) had a superior survival with 92.9% after 1 and 2 years, 83.6% after 3 and 5 years compared to other concepts with 74.4%, 46.1%, 42.5%, and 38.3% after 1, 2, 3, and 5 years, respectively (**Figure 2C**).

Age below or equal to 40 years (p = .002), primary disease (p = .004), chemotherapy in analogy to the EURAMOS-1 trial regimen (any timepoint in treatment, any number of cycles applied) (p = .005), CIBRT (p = .006), and absence of macroscopic residual tumor (p = .036) were identified as prognostic factors for superior OS on univariate analysis (**Table 2**; **Figures 2D–F**). On multivariate analysis, the concept of CIBRT (hazard ratio [HR] = 0.107, 95% confidence interval [CI] = 0.014–0.797, *p* = 0.029) and the absence of macroscopic residual tumor (HR = 0.130, 95% CI = 0.023–0.724], *p* = 0.020) were confirmed as independent predictors of OS.

**Table 2:** Univariate analysis of factors influencing OS.

	N	1-year OS %	2-year OS %	5-year OS %	p=
**All**	49				
**Sex**					.993
Male	26	87.3	66.2	49.7	
Female	23	73.7	54.5	54.5	
**Age**					**.002**
≤40a	29	96.3	73.2	68.3	
**>**40a	20	58.4	42.5	29.8	
**Secondary OS**					.264
No	37	82.9	63.7	52.9	
Yes	12	73.3	48.9	24.4	
**Primary vs. recurrence**					**.004**
Primary	35	85.2	71.6	63.2	
Recurrence	14	68.4	29.3	19.5	
**Biopsy vs. resection**					.781
Biopsy	16	73.7	58.9	44.2	
Any previous resection	33	83.8	61.5	53.8	
**Chemotherapy regimen**					**.002**
EURAMOSS	22	95.5	78.1	72.1	
EURO-B.O.S.S.	9	44.4	33.3	22.2	
Other	8	85.7	42.9	42.9	
None	10	77.8	55.6	44.4	
**Primary plan CTV volume**	47				.173
≤193 cc	25	87.5	63.2	63.2	
>193 cc	22	72.0	54.7	35.3	
**RT concept**					**.009**
CIBRT (OSCAR)	17	92.9	92.9	83.6	
Proton only	3	100	100	100	
Carbon only	12	75.0	41.7	25.0	
Carbon plus IMRT	5	60.0	20.0	20.0	
Carbon Re-RT	12	64.8	51.9	51.9	
**Macroscopic residual tumor**					**.036**
Yes	38	74.4	51.4	43.2	
No	11	100	88.9	77.8	
**Intracranial Infiltration**					.093
Yes	29	88.9	71.9	57.7	
No	20	68.8	42.1	42.1	

Bold values denote statistical significance at the p < 0.05 level.

Information on local progression-free survival (PFS) was available for 45 patients. The median local follow-up of those without local PFS was 24.5 months (range 1–116 months). Local PFS at 1, 2, and 3 years was 67.0%, 57.0%, and 53.6%. After a median local follow-up of 34 months (range 2–87 months) in patients treated with CIBRT, four patients developed local progression; the local PFS at 1, 2, and 3 years was 77.4%.

Information on distant control was limited. For 24 patients, we had no information on serial thoracic CT scans. Of those who progressed, six patients had distant and local progression (mainly pulmonary), two only distant progression, and eight only local progression. Additional four patients died according to the resident’s registration offices: in those cases, we do not have information of local or distant progression previous to death.

At the end of radiotherapy, we observed no acute toxicity higher than grade III (**Table 3**). Grade III toxicity was observed in 9 patients, consisting of mucositis (*N* = 2), dermatitis (*N* = 2), pain (*N* = 4), and increased intracranial pressure (*N* = 1). Of all, 86% of patients were treated as outpatients while 14% required inpatient admission.

**Table 3:** Acute toxicity of patients with craniofacial osteosarcoma .

Acute toxicity	All grades	°I (thereof °I-°II)	°II (thereof °II-°III)	°III
**Nervous system**				
Visual impairment	2	2		
Increased intracranial pressure	1			1
**Skin, appendages, and mucosa**				
Dysphagia	1	1		
Mucositis	26	10 (1)	14 (1)	2
Radiodermatitis	35	29 (5)	4 (1)	2
Hyperpigmentation	2	2		
Conjunctivitis	5	5		
Xerostomia	8	8		
Dysgeusia	5	3	2	
Xerophthalmia	3	2	1	
Dry nasal mucosa	1		1	
Epistaxis	2	2		
Alopecia (focal)	12	10	2	
**Ear and labyrinth**				
Otorrhea/Otitis externa	3		3	
Middle ear fluid	2		2	
Dizziness	3	3		
Tinnitus	2	2		
**Other**				
Fatigue	7	7		
Nausea	3	2	1	
Pain	12	5	3	4
Lymph edema (periorbital)	6	5	1	
Hypesthesia	1	1		

Information on late toxicity was available for *N* = 23 patients (**Table 4**). It included central nervous system necrosis °II in three patients with initial intracranial extension of tumor: two were successfully treated with dexamethasone and one with bevacizumab. Another patient with initial intracranial tumor extension in proximity to the temporal lobe developed epilepsy (°II) successfully treated by medication and hearing loss due to infiltration of petrous bone which required a contralateral cochlear implant because of bilateral otosclerosis. Buccal fistula was observed in a patient who had undergone three attempts of tumor resection within 2 months before radiotherapy and a reconstruction of the maxilla that was performed after the end of RT. One patient with an extensive intracranial infiltration of the frontal lobe developed an empyema shortly after RT and required surgery; whether this infection was facilitated by radiotherapy or is purely tumor associated cannot be differentiated with certainty. Lastly, a patient treated with RT at the age of 9 after subtotal resection of an osteosarcoma infiltrating the maxilla, orbit, and the paranasal sinuses presented with a facial asymmetry with a hypoplastic midface, ipsilateral open bite, and persisting milk teeth of the upper jaw at the age of 18; reconstructive surgery is planned in future.

**Table 4:** Late toxicity of patients with craniofacial osteosarcoma (n = 23).

Late toxicity	All grades	°I	°II	°III	°IV
**Nervous system**					
Visual impairment	1	1			
Intracranial empyema	1			1	
Anosmia	2	2			
Dysgeusia	4	3	1		
CNS necrosis	7	4	3		
Concentration difficulties	1	1			
Epilepsy	1		1		
**Skin, appendages, and mucosa**					
Xerostomia	4	1	3		
Dry nasal mucosa	1		1		
Foetor	1	1			
Alopecia (focal)	5	3	2		
Epiphora	1	1			
Chronic radiodermatitis	1	1			
**Head and neck**					
Sinusitis	1	1			
Trismus	1	1			
**Ear and labyrinth**					
Dizziness	1	1			
Tinnitus	1		1		
Hearing impairment	1				1
**Other**					
Fatigue	3	3			
Pain	3	3			
Pituitary gland impairment	1		1		
Buccal fistula	1		1		
Lymph edema	1	1			
Midfacial hypoplasia	1	1			

Chi-square test was used to test statistical association. Statistical significant *P*-values are shown in bold.

## Discussion

In this study, we present the results of the largest CFOS cohort treated by particle therapy thus far. Our results show that in cases where surgery fails to achieve and maintain local control, different approaches of particle therapy offer a treatment alternative with promising results. The estimated 3-year local PFS was 53% and 5-year OS was 51%. Moreover, the toxicity of the RT was limited.

The German–Austrian–Swiss osteosarcoma study group reported treatment results of 49 CFOS patients treated from 1977 to 2004. Here, complete surgical remission was achieved in 32 patients, of whom 24 remained in long-term local control. A 5-year OS of 74% and an event-free survival (EFS) of 44% were reported by this study. Extragnathic site and documented postoperative rest of the primary tumor were associated with impaired survival. Two-year OS was only 38.8% for the 13 patients with a documented tumor rest. Of these, six patients received additional radiotherapy with a lower total dose than in the current study (50–62.5 Gy) (2).

König et al. investigated a cohort of 42 CFOS patients treated with surgery at the Oslo University Hospital. Here, inoperable patients were excluded. In line with the current cohort, the addition of chemotherapy resulted in better survival. The postoperative OS rate was 70.5% at 2 years and 44.7% at 5 years and disease-specific survival after non-radical surgery was 65.0% and 39.3% after 2 and 5 years compared to 86.7% and 66.7% after adequate surgery. Radiotherapy was administered in 60% of patients as an adjunct to suboptimal resection or against recurrence or metastases; the total dose was also here lower with a median of 60 Gy, and RT did not show a significant correlation to OS (11). In another cohort of 119 patients, 23 were treated with a combination of surgery and radiotherapy regardless of whether the resection margins were positive or negative. The median total dose was a 60 Gy (range, 50–66 Gy); for those with positive or uncertain resection margins, RT resulted in superior local control (75% vs. 24%). The median OS was at 63% at 5 years and 55% at 10 years (12). The three aforementioned studies are not entirely comparable to the present study as they mostly address resection margins but not inoperable disease. Hence, our cohort probably consists of the more advanced cases. Still, the local control and OS are comparable to those studies and we hypothesize that this could be explained by the significantly higher total RT dose applied in the treatment of the present cohort. It is likely that a dose below 70 Gy (equivalent dose in 2 Gy fractions) might not be sufficient to achieve local control in CFOS.

Data on particle therapy for CFOS are available from the US (13, 14). Fifty-five patients with osteosarcoma were treated with protons only (20%) or with a combination with photons (80%); 22 of these had CFOS. For the whole cohort, the mean radiation dose was 68.4 Gy and the mean CTV volumes for the primary and boost plan were 213 and 82 cc, respectively. Unfortunately, radiation dose or survival rates were not reported separately for the CFOS cohort (13). Disease-free survival and OS at 5 years were 65% and 67%, respectively. Localization in the skull was associated with a higher risk for local failure (HR 2.6); the cumulative incidence of local failure was 25% after 1 year.

In the current study, CIBRT was superior compared to the carbon ion only or carbon ion plus IMRT concept. Indeed, OS of 92.9% after 2 years and 83.6% after 5 years are highly promising. We assume that the strict definition of the target volume delineation especially in regard to CTV margins and fixed prescription dose for the base and boost of the OSCAR protocol contributed to the superior results. The CTV margins and total dose of other treatment regimens were more varied and often based on the treating physician choice. Due to the small patient numbers, it is additionally possible that the influence of factors such as age, chemotherapy, and recurrence vs. primary disease might be underestimated on multivariate analysis. Especially age differed, when comparing the groups carbon ion only and CIBRT. Patients treated by carbon ions only were more often above the age of 40 years compared to patients treated by CIBRT.

In a systematic review, the latency between radiotherapy for retinoblastoma and the onset of secondary craniofacial sarcomas was 12 years. The mean overall survival for secondary osteosarcoma was 20 months (14–32) and significantly worse in cases with cranial extension (15). Secondary CFOS behaved more aggressively and demonstrated higher expression rates of adverse prognostic factors (overexpression of p53, higher proliferative activity) (16). Treatment of secondary osteosarcoma remains challenging. Due to the foregoing irradiation, total dose and CTV margins applied here were lower compared to the remaining cohort.

While this is the largest cohort on particle therapy for craniofacial osteosarcoma to date, the study has some notable limitations. The HIT is a highly specialized center, and most patients in this cohort traveled long distance sometimes even across borders to receive treatment. Understandably, several decided to perform the follow-up visits closer to their home for their long-term monitoring. For those who did not present at our center, we offered additional revision of local MRIs and thoracic CT scans. In addition, attempts were carried out to update information on toxicity, survival, and local and distant control from outside centers by contacting local oncologists and referring physicians. Nonetheless, the median local and distant follow-up times were significantly lower compared to overall survival and there is substantial risk of underreporting late toxicity. Additionally, different chemotherapy regimens were used and also the timepoint of chemotherapy and the exact number of cycles differed.

Historically, osteosarcoma has been considered as radioresistant, and RT has not played an important role in the treatment of these tumors. The development of highly conformal treatment techniques such as particle therapy resulting in dose escalation to the tumor and simultaneous reduction of dose to the adjacent organs at risk has generated a promising alternative for local treatment. Our data show that for inoperable or incompletely resected CFOS, particle therapy and especially CIBRT should be considered. Further investigations are needed in order to establish a treatment regimen for this extremely urgent condition and provide more reliable data.

## Data availability statement

The raw data supporting the conclusions of this article will be made available by the authors, without undue reservation.

## Ethics statement

This study was reviewed and approved by the Ethics committee of the University of Heidelberg. Written informed consent for participation was not required for this study in accordance with the national legislation and the institutional requirements.

## Author contributions

KS, KH, JD, SH contributed the conception and design of the study. KS and MD analyzed the data. JL, SA, FW, MS, CF, JH, IH, provided the data, KS, IH, SH wrote the manuscript. All authors contributed to manuscript revision, read and approved the submitted version.

## Funding

For the publication fee we acknowledge financial support by Deutsche Forschungsgemeinschaft within the funding programme “Open Access Publikationskosten” as well as by Heidelberg University.

## Conflict of interest

The authors declare that the research was conducted in the absence of any commercial or financial relationships that could be construed as a potential conflict of interest.

## Publisher’s note

All claims expressed in this article are solely those of the authors and do not necessarily represent those of their affiliated organizations, or those of the publisher, the editors and the reviewers. Any product that may be evaluated in this article, or claim that may be made by its manufacturer, is not guaranteed or endorsed by the publisher.
